# Supplementary material for: Ice shelf basal channel shape determines channelized ice-ocean interactions
Source: Nat Commun. 2024 Apr 3;15:2877. doi: 10.1038/s41467-024-47351-z (PMC10991488; doi:10.1038/s41467-024-47351-z)
Supplement: Supplementary file 1 — Supplementary Information [file 41467_2024_47351_MOESM1_ESM.pdf]

Supplementary information for the manuscript:

Ice shelf basal channel shape determines channelized ice-ocean interactions

Chen Cheng[1], **Adrian Jenkins**[†2], **Paul R. Holland**[†3], **Zhaomin Wang**[1], **Jihai Dong**[4,1], **Chengyan Liu**[1]

[1]Southern Marine Science and Engineering Guangdong Laboratory (Zhuhai), Zhuhai, 519080, China

[2]Department of Geography and Environmental Sciences, Faculty of Engineering and Environment, Northumbria University, Newcastle upon Tyne, NE1 8ST, UK

[3]British Antarctic Survey, Cambridge, CB3 0ET, UK

[4]School of Marine Sciences, Nanjing University of Information Science and Technology, Nanjing, 210044, China

Correspondence to:

Chen Cheng

chengchen@sml-zhuhai.cn

[†]These authors contributed equally to this work.

This file contains:

Supplementary Figures 1-5

Supplementary Note 1 including Supplementary Table 1

Supplementary Note 2 including Supplementary Figures 6-8

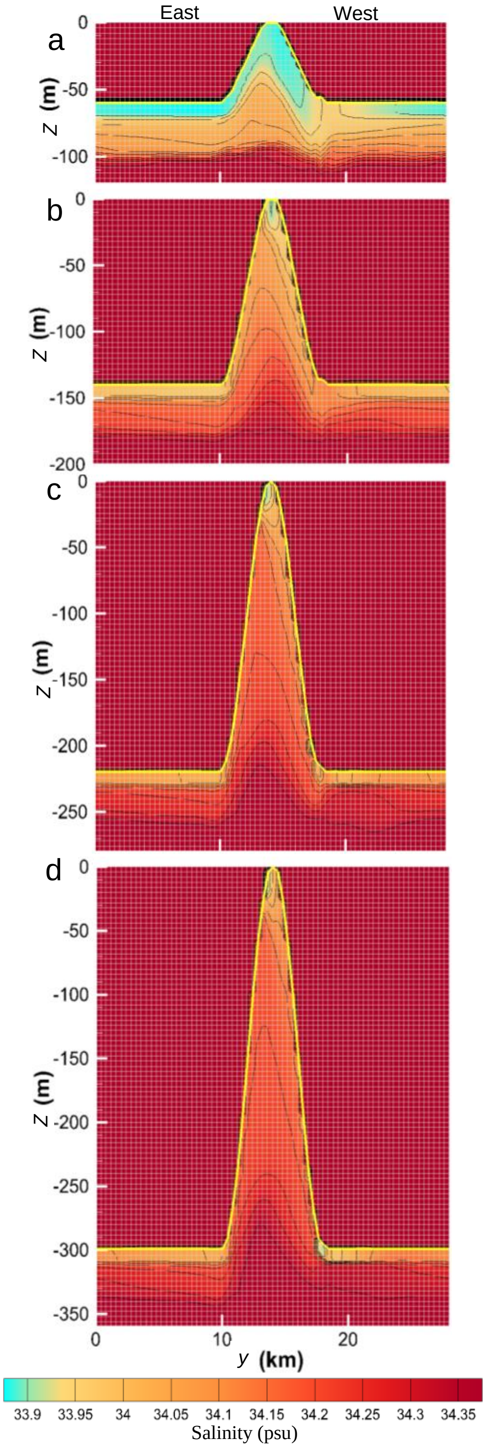

Supplementary Figure 1. Time-averaged vertical haline structure in the cross section at the northern boundary in a H60W8, b H140W8, c H220W8, and d H300W8. The ice base in each plot is marked by the yellow line, and the planar part of ice base corresponds to the same ice draft. The default value of 34.36 psu inside the solid ice (i.e., above the planar ice base and outside the channel region) has no physical significance. Model grids are also indicated by the mesh.

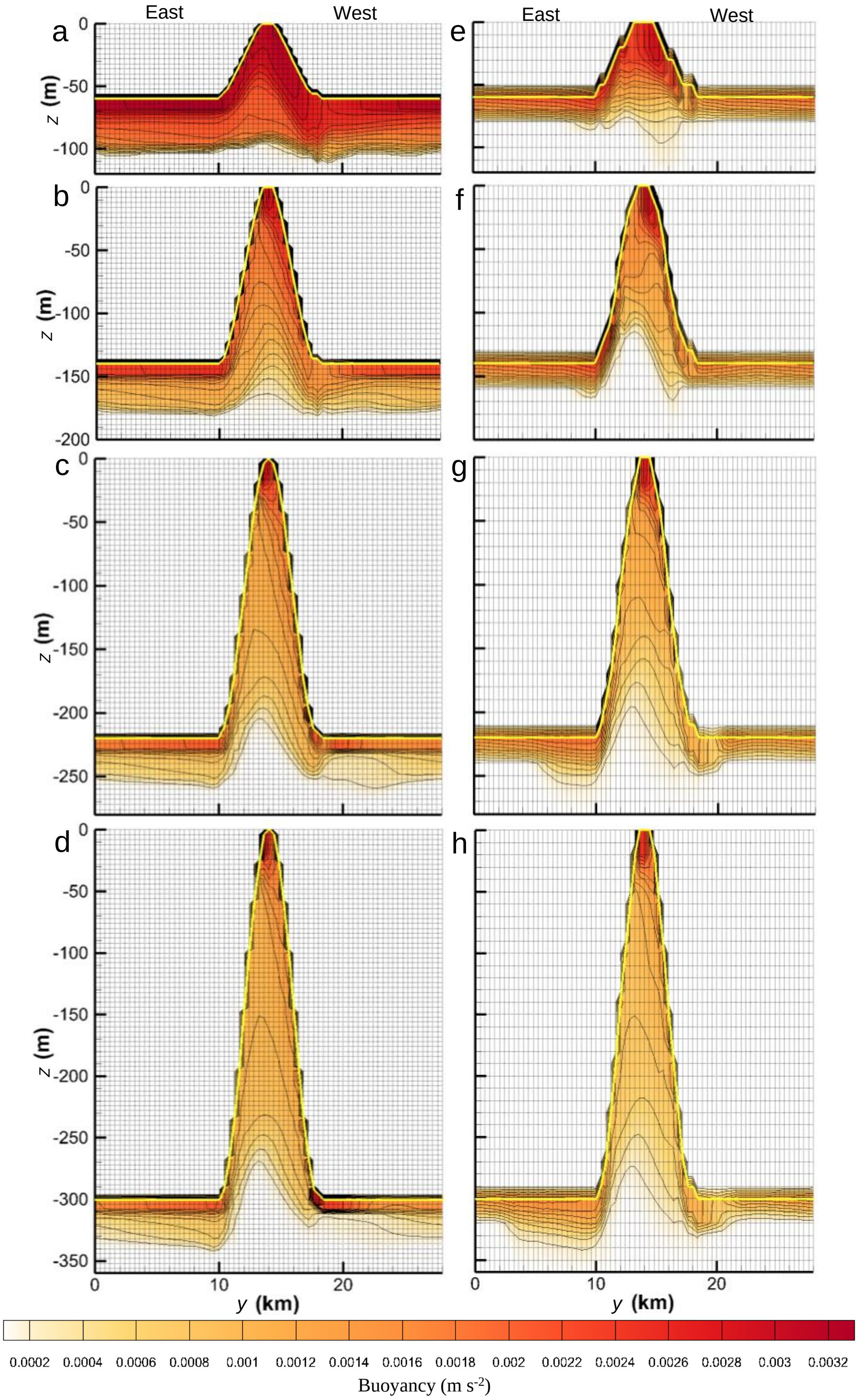

Supplementary Figure 2. Time-averaged stratification in the cross section at the northern boundary in a H60W8, b H140W8, c H220W8, and d H300W8. e-h are for the corresponding runs with coarsened vertical resolution (10 m). The ice base in each plot is marked by the yellow line, and the planar part of ice base corresponds to the same ice draft. Model grids are also indicated by the mesh.

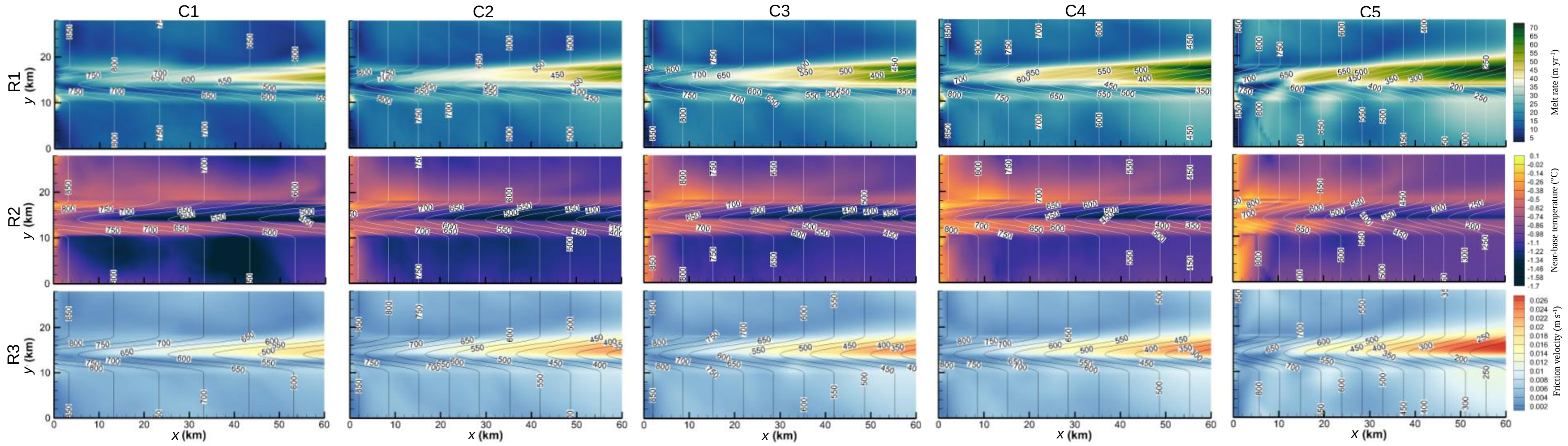

Supplementary Figure 3. Differential basal melt and its determinants caused by differential basal slope and ambient water properties. Distribution of (R1) basal melt rates, time-averaged (R2) near-ice temperature and (R3) friction velocity in (C1) Sp05, (C2) Sa34.31, (C3) H140W8, (C4) Sa34.41, and (C5) Sp11. The white and black contour lines in R1, 2 and R3, respectively, indicate the ice shelf draft.

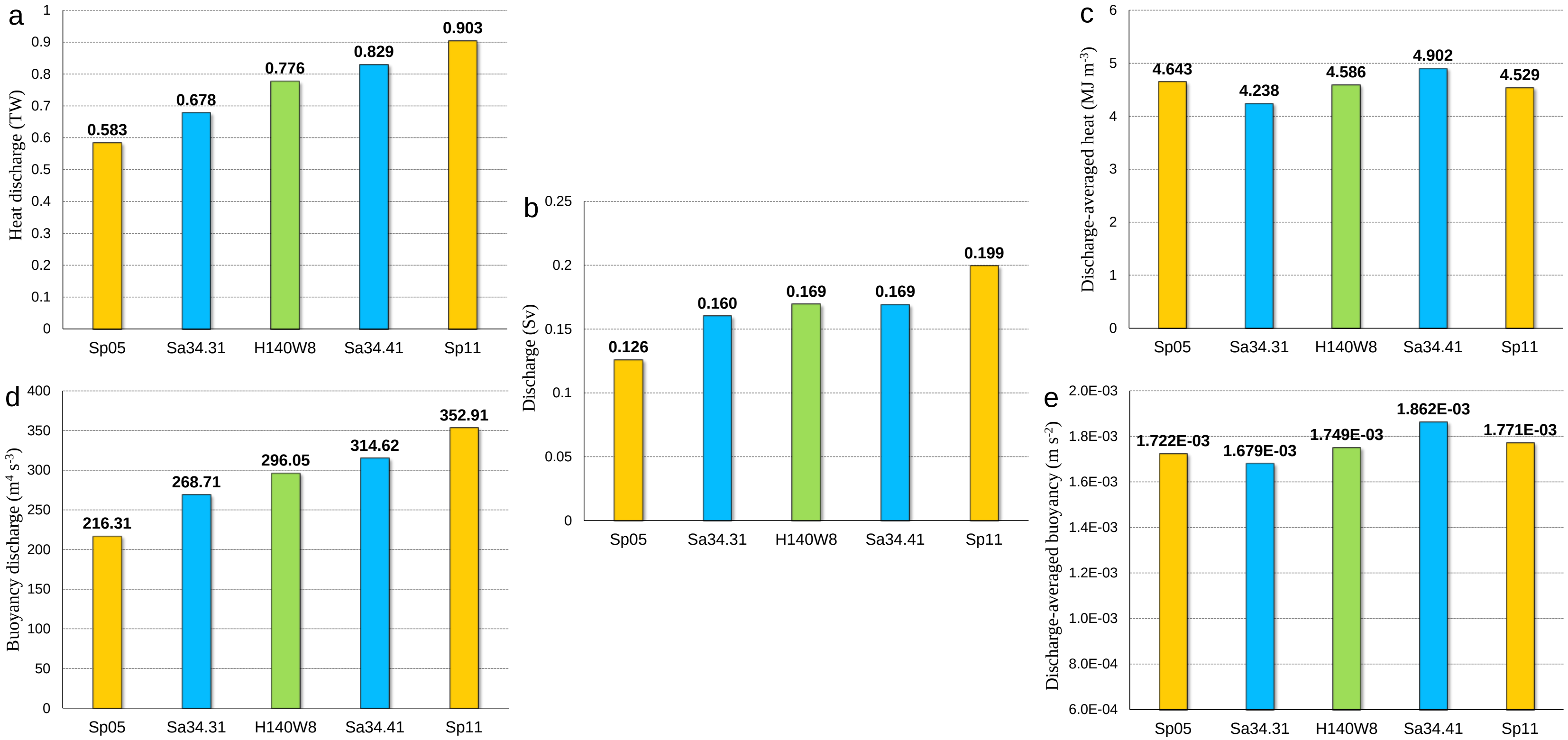

Supplementary Figure 4. Comparison of time-averaged quantities relevant to channelized outflow for runs in S2. a Heat discharge; b discharge; c discharge-averaged heat; d buoyancy discharge; e discharge-averaged buoyancy.

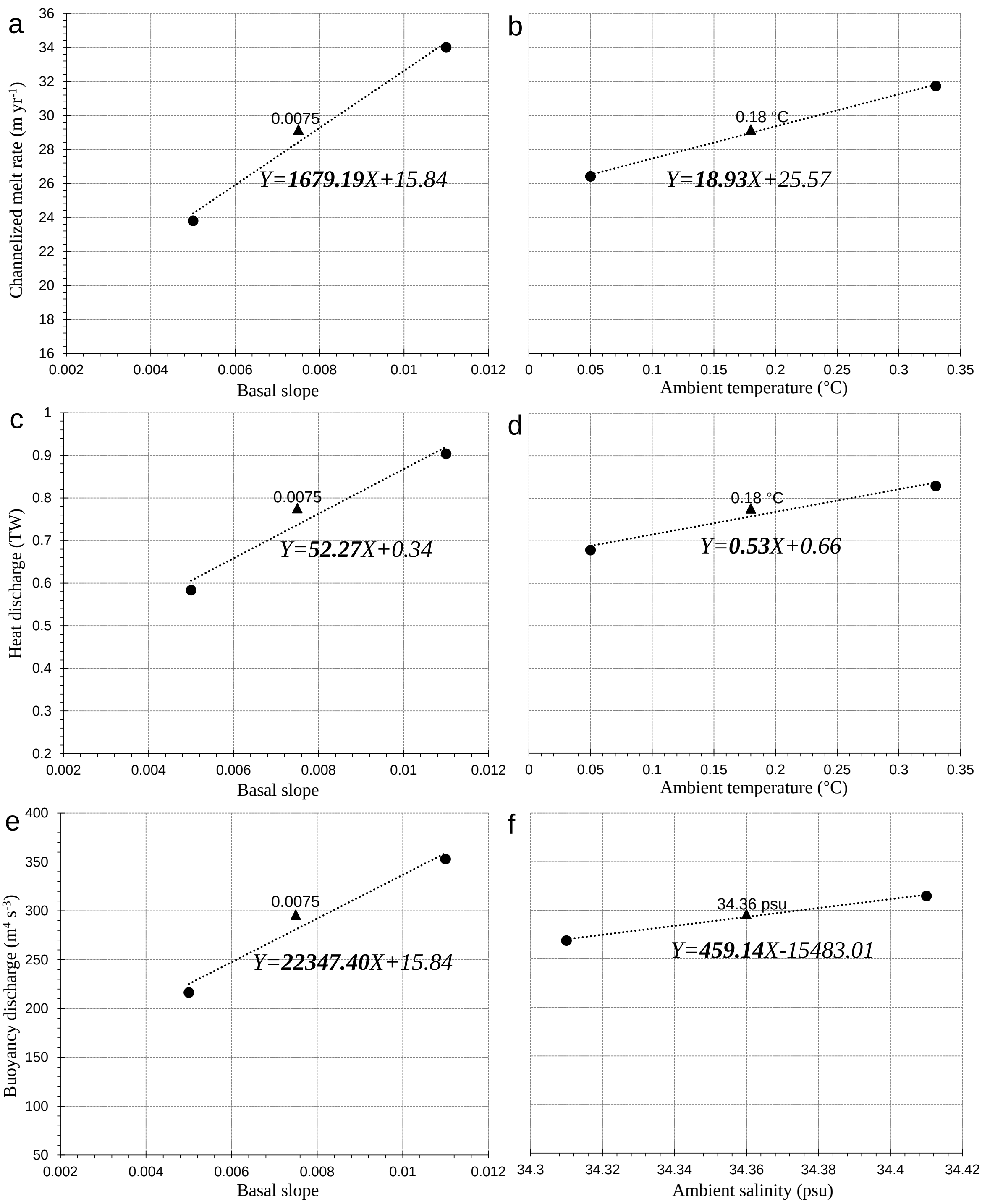

Supplementary Figure 5. Linear relationship between channelized quantities and varying factors in S2. Dependent variables are channelized a, b basal melt rate, c, d heat discharge, and e, f buoyancy discharge. Independent variables of a, c, e ice shelf basal slope, b, d ambient temperature, and f salinity. In each plot, the value of the corresponding independent variable in H140W8 (solid triangle) is labeled, and the slope of the regression line is highlighted.

Supplementary Note 1 Limitation of the current resolution

Given the imperfect representation of the channel CSS using the standard resolution (dy=400 m, dz=4 m), we examine the reliability of the derived relationships between channelized quantities and channel CSS by performing higher resolution (dy=250 m and/or dz=2 m) simulations for the shallowest (60 m) and the narrowest (4 km) cases. Six additional runs, including H60W8 and H60W12 with dz=2 m, H60W4 with both dy=250 m and dz=2 m, and H140W4, H220W4, and H300W4 with dy=250 m, have been conducted, and the statistics of the simulated channelized quantities are summarized in Supplementary Table 1. For the narrowest channels, excluding H60W4 where the across-slope resolution increases by 60%, the maximal deviations of the channelized melt rate and discharge quantities in the higher resolution are 11% and 21%, respectively. The deviations for the shallowest cases with wider channels are even less, except for H60W4 that has the largest deviation among all these six runs, suggesting that the empirical relationships developed here should not be used for basal channels close to the lower limit of cross-sectional area.

Supplementary Table 1 | Deviation from higher resolution simulations

Nomenclature	(dy, dz) (m)	Channelized quantities				(dy, dz) (m)	Channelized quantities				Deviation (%)			
		M (m yr^{-1})	D (Sv)	HD (TW)	BD (m^4 s^{-3})		M (m yr^{-1})	D (Sv)	HD (TW)	BD (m^4 s^{-3})	M	D	HD	BD
H60W12	(400, 4)	22.3	0.112	0.302	302.92	(400, 2)	22.7	0.115	0.346	297.14	**1.9**	**2.9**	**12.7**	**1.9**
H60W8		22.8	0.081	0.218	221.05	(400, 2)	22.1	0.080	0.231	210.11	**2.8**	**1.9**	**5.8**	**5.2**
H60W4		22.0	0.042	0.106	118.18	(250, 2)	18.0	0.032	0.076	95.53	**22.7**	**30.0**	**40.7**	**23.7**
H140W4		24.1	0.094	0.412	177.83	(250, 4)	24.6	0.085	0.357	166.42	**1.9**	**11.0**	**15.6**	**6.9**
H220W4		27.1	0.152	0.810	215.21	(250, 4)	27.7	0.126	0.675	176.98	**2.4**	**20.7**	**20.0**	**21.6**
H300W4		30.0	0.200	1.194	221.18	(250, 4)	33.6	0.189	1.124	207.73	**10.9**	**5.7**	**6.2**	**6.5**

Deviation (bold figures) is defined here as the difference between the cases with standard and higher resolution expressed as a percentage of the higher resolution case. M, D, HD, and BD denote melt rate, discharge, heat discharge, and buoyancy discharge, respectively.

Supplementary Note 2 Deviation induced by coarse vertical resolution

The purpose of the S3 experiments is to quantify the influence of model vertical resolution, 4 m vs 10 m, on channelized melt and transport. This is motivated by the work of Millgate et al.[26], who used a 3D ice-cavity model with a vertical resolution of 10 m. Adopting the coarse vertical grid leads to greater and more extensive channelized melt (Supplementary Fig. 6-C1, 2), which arises primarily from higher near-ice temperature (Supplementary Fig. 6-C3, 4), while differences in the friction velocity are less noticeable (Supplementary Fig. 6-C5, 6). Use of the coarse vertical grid can also lead to larger heat (Supplementary Fig. 7a) and lower buoyancy discharge (Supplementary Fig. 7b), differences that arise consistently from changes in the discharge-averaged heat (Supplementary Fig. 7d) and buoyancy (Supplementary Fig. 7e), both of which decrease as the basal channel deepens. Differences in the channelized discharge are not significant, except for the runs with the shallowest (60 m) basal channel (Supplementary Fig. 7c). Overall, the adoption of coarser vertical resolution artificially generates warmer and saltier water masses inside the channel. This can be explained by the resultant differences in the stratification (Supplementary Fig. 2) and dynamic processes (Supplementary Fig. 8).

The GMW plume and its underlying pycnocline structure cannot be reproduced in the shallowest channel (Supplementary Fig. 2a) using the inferior vertical resolution (Supplementary Fig. 2e), giving rise to the largest thermohaline differences indicated in Supplementary Fig. 7d and e. In H60W8-C, except for the main channel flow area, the near-ice Ekman layer cannot be resolved at all (Supplementary Fig. 2e), resulting in the low vertical diffusivity (Supplementary Fig. 8a). In addition, the steeper vertical gradients at the upper and lower interfaces of the underlying pycnocline cannot be reproduced, and instead merge into an overly-smooth single pycnocline (Supplementary Fig. 2e) that results in exaggerated vertical diffusion (Supplementary Fig. 8a), compared with that in H60W8 (Fig. 6a). When the channel is deepened to 140 m (H140W8-C), the near-ice Ekman layer still cannot be resolved for the regions outside the main channel flow (Supplementary Figs. 2f and 8b). The upper interface of the pycnocline can be formed (Supplementary Fig. 2f) but is much less tilted than that in H140W8 (Supplementary Fig. 2b). The topographic secondary overturing emerges but is weaker (Supplementary Fig. 8f) than that in H140W8 (Fig. 6f), because the steepness of the isopycnals is much

reduced with the larger vertical spacing (Supplementary Fig. 2b, f). Moreover, the stratification associated with the recirculation of iGMW (Supplementary Fig. 2b) is markedly reduced in H140W8-C (Supplementary Fig. 2f), resulting in an overestimated vertical diffusivity along the western channel flank (Supplementary Fig. 8b). When the channel is deepened further, the stratification (Supplementary Fig. 2g, h) and dynamic processes (Supplementary Fig. 8c, d, g, h) for the coarser vertical resolution progressively approach that for the higher vertical resolution (Supplementary Fig. 2c, d and Fig. 6c, d, g, h), including the emergence of a still poorly resolved near-ice Ekman layer to the east of the channel. This gradual improvement in the representation of deeper channels accounts for the decrease in the differences in discharge-averaged heat and buoyancy discussed above.

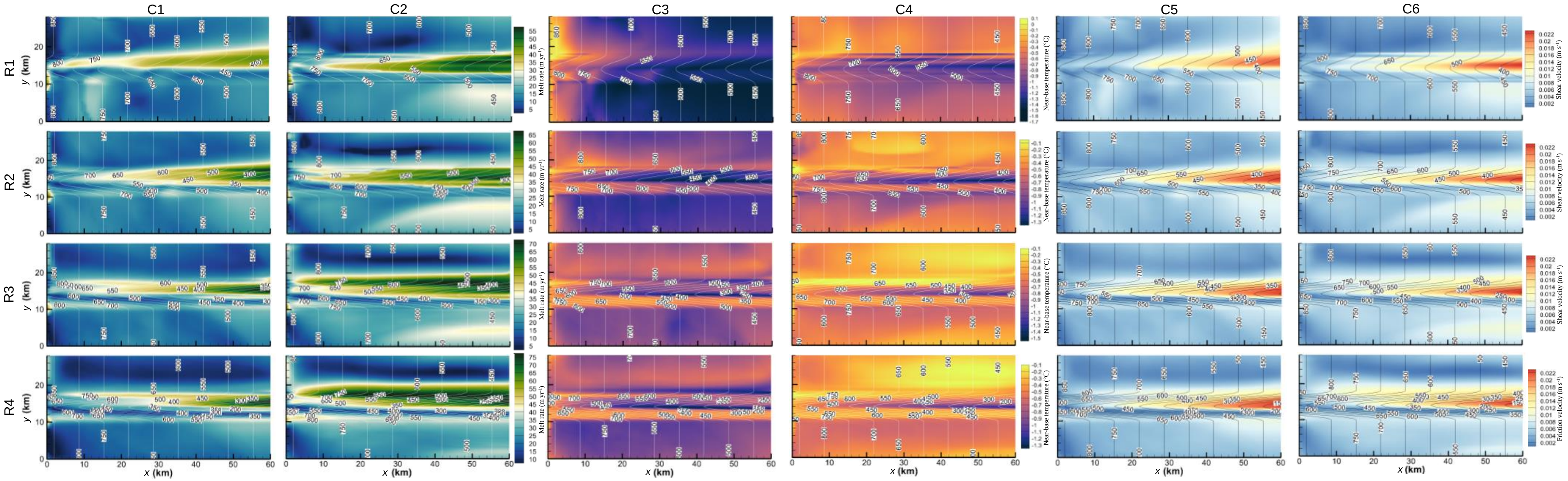

Supplementary Figure 6. Differential basal melt and its determinants caused by coarser vertical resolution. Distribution of (C1, 2) basal melt rates, time-averaged (C3, 4) near-ice temperature, and (C5, 6) friction velocity in (R1; C1, 3, 5) H60W8, (R1; C2, 4, 6) H60W8-C, (R2; C1, 3, 5) H140W8, (R2; C2, 4, 6) H140W8-C, (R3; C1, 3, 5) H220W8, (R3; C2, 4, 6) H220W8-C, (R4; C1, 3, 5) H300W8, and (R4; C2, 4, 6) H300W8-C. The white and black contour lines in C1-4 and C5, 6, respectively, indicate the ice shelf draft.

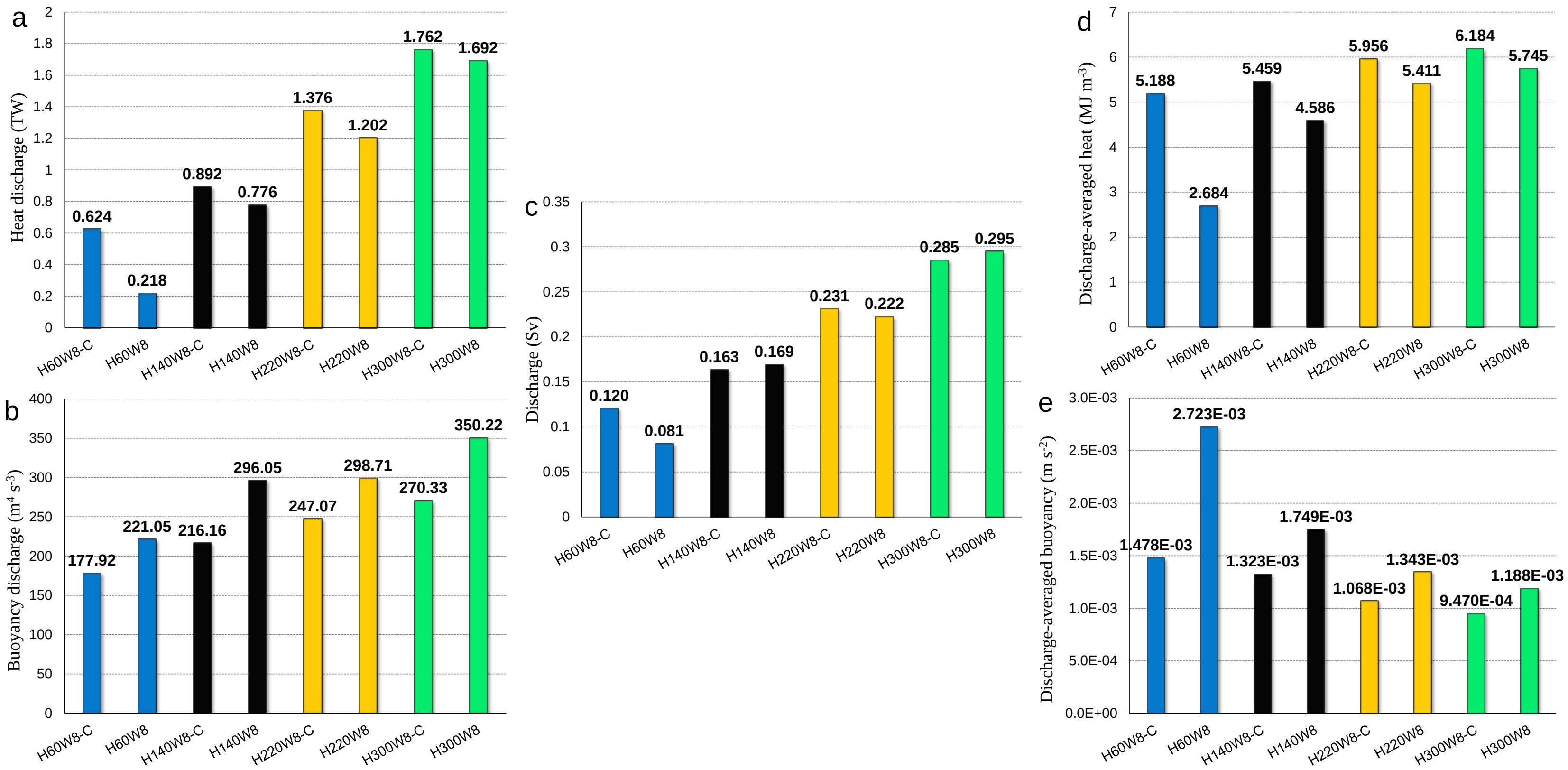

Supplementary Figure 7. Comparison of time-averaged quantities relevant to channelized outflow for runs in S3 and their counterparts in S1. a Heat discharge; b buoyancy discharge; c discharge; d discharge-averaged heat; e discharge-averaged buoyancy.

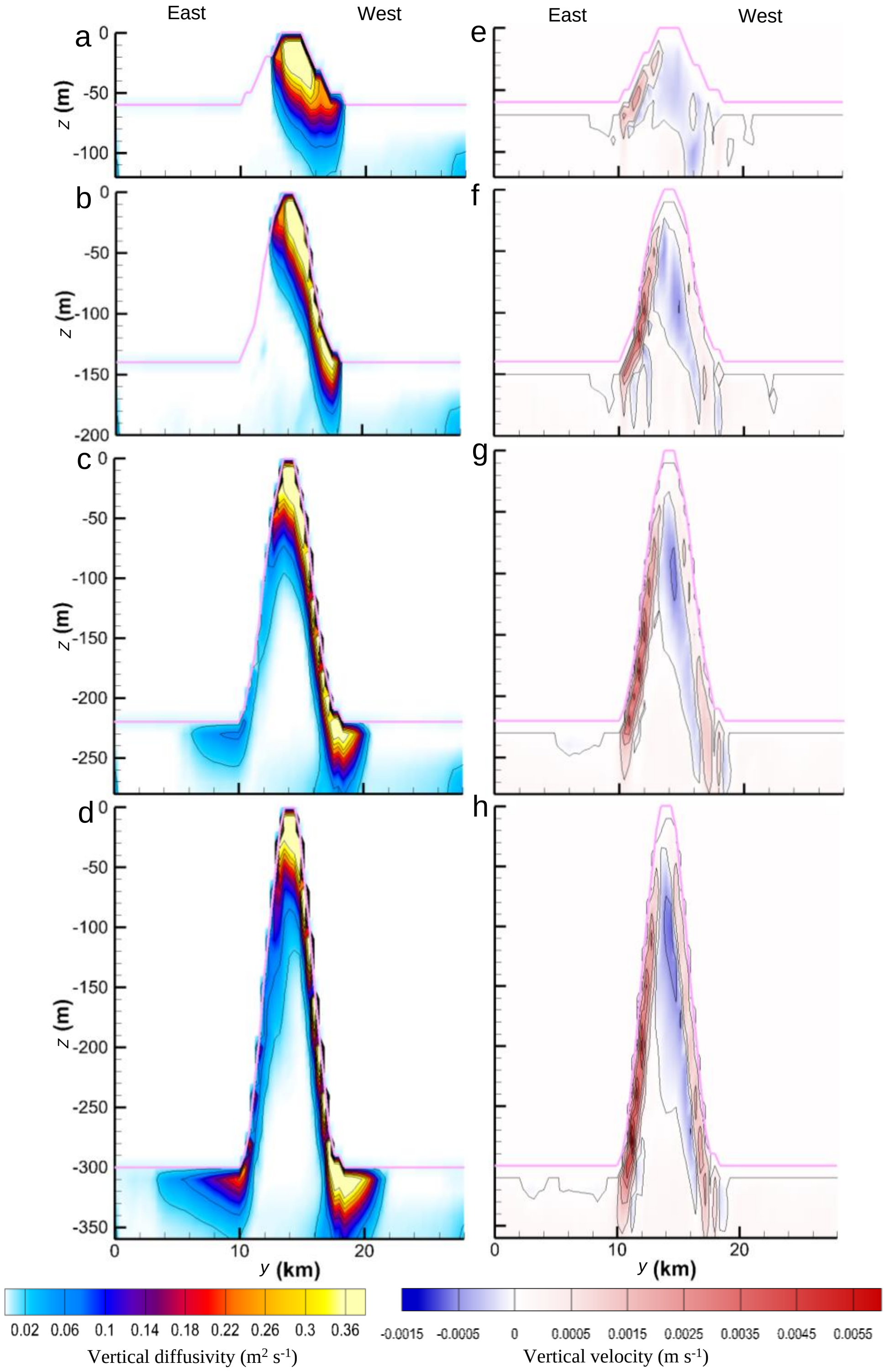

Supplementary Figure 8. Variables associated with channelized vertical heat transport for S3 runs. Section-averaged (i.e., x=30 and 60 km) across-slope distribution of time-averaged a-d vertical diffusivity and e-h vertical velocity in a, e H60W8-C, b, f H140W8-C, c, g H220W8-C, and d, h H300W8-C. The ice base in each plot is marked by the pink line, and the planar part of ice base corresponds to the same ice draft.
